# Anti-bacterial activity of baicalin against APEC through inhibition of quorum sensing and inflammatory responses

**DOI:** 10.1038/s41598-019-40684-6

**Published:** 2019-03-11

**Authors:** Lu-Yuan Peng, Meng Yuan, Zong-Mei Wu, Ke Song, Chun-Lei Zhang, Qiang An, Fang Xia, Jia-Lin Yu, Peng-Fei Yi, Ben-Dong Fu, Hai-Qing Shen

**Affiliations:** College of Veterinary Medicine, Jilin University, No. 5333 Xi’an Road, Changchun, Jilin 130062 China

## Abstract

Avian pathogenic *Escherichia coli* (APEC), collectively known as causative agent of extraintestinal infections, is an important cause of morbidity and mortality in poultry. Currently, quorum sensing (QS), biofilm formation and virulence factors are considered as novel prospective targets for antimicrobial therapy to control APEC invasion. In addition, inflammatory responses are also served as the major pathological features of APEC invasion. This study was aimed to explore the effect of baicalin on APEC and APEC-induced inflammatory responses. After treatment with baicalin, we mainly examined the AI-2 secretion, biofilm formation, expression of virulence genes of APEC, and the levels of inflammatory cytokines, as well as the expression of NF-κB pathway. Our results showed that baicalin significantly inhibited the QS via decreasing the AI-2 secretion, biofilm formation, and the expression of virulence genes of APEC such as *LsrB, LsrK, LuxS, pfs, H-NS, fimA, fimB, fyuA, csgA, csgB*, and *rpoS*. Moreover, baicalin significantly attenuated the release of lactate dehydrogenase (LDH), and the adhesion of APEC to chicken type II pneumocytes to reduce cell damage. Furthermore, baicalin also inhibited the expression of pro-inflammatory cytokines and NF-κB activation. Thus, our data revealed that baicalin could interfere with the quorum sensing, biofilm formation and virulence genes expression to relieve the APEC pathogenicity. Additionally, baicalin decreased the inflammatory responses of chicken type II pneumocytes induced by APEC. Taken together, these data provide a novel potential pharmaco-therapeutic approach to chicken colibacillosis.

## Introduction

Chicken colibacillosis, as one of the principal causes of morbidity and mortality in poultry worldwide, is characterized by multiple organ lesions such as air sacculitispericarditis, peritonitis, salpingitis, synovitis, osteomyelitis or yolk sac infection, and caused by a group of pathogens designated avian pathogenic *Escherichia coli* (APEC)^1,2^. Quorum sensing (QS) is a widespread signaling system that controls the responses of bacterial populations to cell density, consisting of signal molecules (autoinducers), signal synthases, and signal receptors^3^_._ The signal molecules mainly includes autoinducer 1 (AI-1) and autoinducer 2 (AI-2). Of which, the AI-2 QS system is widely present in most gram-negative and gram-positive bacteria and has been proved to regulate the gene expression and physiological behaviors of bacteria in either intraspecies or interspecies communication^4^. The pathogenicity of APEC is also regulated by QS systems, and AI-2 regulates the expression of genes involved in various processes, including secretion of virulence factors, biofilm formation, motility, genetic competence, sporulation, and antibiotic production^5,6^. APEC has plentiful virulence factors, including QS signal molecule synthesis genes (*LuxS* and *pfs)*, transported and phosphorylated genes(*LsrB* and *LsrK)*, bacterial survival stress related genes (*rpos* and *H-NS)*, iron uptaking related genes (*IucD* and *fyuA*), type 1 pili (*fimA* and *fimB*), and curly pili A (*csgA* and *csgB*)^7^. Han has reported the *luxS* mutant of APEC showed a reduced bacterial mortility and decreased mRNA levels of the virulence-related genes^8^. Some findings have suggested that the identified AI-2 inhibitors possess anti-biofilm effects against APEC- O78 likely through the down-regulation of genes associated with adhesion, motility, and capsule synthesis among others^5,9^. Meanwhile, the biofilm, surface-associated bacterial communities embedded in an extracellular matrix, protects against host immune reactions or antibiotics, and is a major problem in the context of chronic infection^10^.

In addition, the inflammatory response is considered to be the first defense line against the pathogenic invasion^11^. The pathogenic stimulation will lead to the production of a large number of pro-inflammatory cytokines, including TNF-α, IL-1β, and so on. These up-regulated cytokines cause edema, cellular metabolic stress, and tissue necrosis^12^. IL-1β and IL-6 were significantly increased in mice with sepsis induced by *E. coli*^13^ and the mice treated with *Lepidium sativum* polysaccharides significantly inhibited *E. coli*-induced inflammation by reducing the circulating levels of TNF-α^14^.

QS, biofilm formation and virulence factors contribute significantly to the immune evasion and the tolerance to a variety of antimicrobial treatments, and an vital role on inflammatory response after APEC invasion.

**Figure 1:**
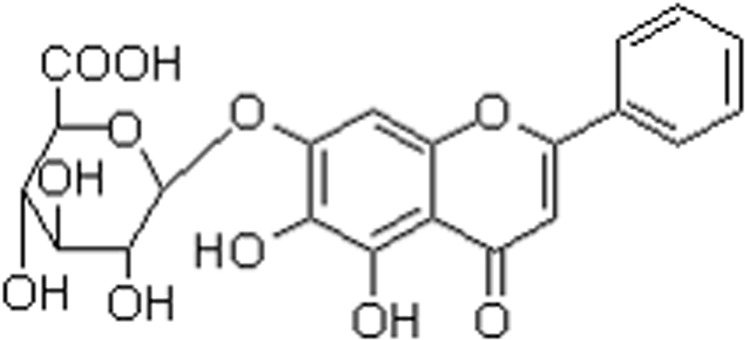
The structure of Baicalin.

The flavonoids have been demonstrated as QS inhibitors to suppress the bacterial cell-cell signaling^15–20^, such as naringenin, kaempferol, quercetin, apigenin, baicalein and so on. Baicalin (Fig. 1), one flavonoid compound extracted from the dried roots of Huangqin, has been proved to possess inhibitory effects on virulence phenotypes regulated by QS in *Pseudomonas* aeruginosa^16^. However, whether baicalin could interfere QS in APEC remains unknown. On the other hand, some recent studies have reported that baicalin can alleviates IL-1β-induced inflammatory injury in chondrocytes^21^, protect against lead-induced renal oxidative damage in mice^22^, and lessen the liver inflammation caused by lipopolysaccharide in chicken^23^. In addition, baicalin reduced age-related inflammation through blocking pro-inflammatory NF-κB activation^24^. Based on this, we investigated the effects of baicalin on QS, biofilm formation, virulence genes expression of APEC and inflammatory responses induced by APEC, aiming to find one new treatment to suppress the chicken colibacillosis.

## Material and Methods

### Reagents and bacterial strains

Baicalin (purity ≥ 98%) was purchased from Chengdu Must Biotechnology Co., Ltd. (Chengdu, China). Luria-Bertani (LB) medium was obtained from Sigma-Aldrich (St.Louis, MO, USA). Fetal bovine serum (FBS) and DMEM were obtained from Gibco (Invitrogen S.r.l., Milan, Italy). TRIZOL reagent and PrimeScriptTM RT Reagent Kit with gDNA Eraser were purchased from TaKaRa (Da Lian, Liaoning, China). All other chemicals were of reagent grade. Chicken mAb phospho-NF-κB p65 antibodies and chicken mAb Phospho-IκBα were purchased from Sangon Biotech Company (Shanghai, China).

### Bacterial strains and growth condition

APEC-O78 strain (CVCC1418) was purchased from Chinese Veterinary Culture Collection Center (CVCC, Beijing, China). The bacteria were grown routinely in LB agar plates and then pick a single colony to LB medium for culturing at 37 °C overnight. The OD_600_ was monitored with a SynergyTM HT Multi-Mode Microplate Reader (BioTek Instruments, Winooski, VT). Vibrio harveyi BB152 (*V. harveyi* BB152) (sensor1+ sensor2+) strain was provided by Dr. Han of Shanghai Veterinary Research Institute (CAAS, Shanghai, China), Vibrio harveyi BB170 (*V. harveyi* BB170) (sensor1− sensor2+) strain was donated by Dr. Ke of College of Ocean & Earth Sciences (Xiamen University, Xiamen, China) and cultivated in modified autoinducer bioassay medium at 30 °C^25^. *E. coli* DH5α (engineering *E. coli*, no pathogenicity) was purchased from Takara Bio Inc.

### Determination of the minimum inhibitory concentration (MIC) of baicalin

The MIC of baicalin against APEC-O78 was determined according to the standard Clinical and Laboratory Standards Institute (CLSI) broth microdilution method. Baicalin was initially dissolved in pure dimethyl sulfoxide (DMSO), and diluted in LB broth to result in final concentration of 12.5, 25 and 50 μg/mL. The bacteria solution at an initial turbidity of 0.05 (OD_600_) were added to the 96-well microtitre plate. Each well contains 50 μL of bacteria and 100 μL of baicalin (final concentrations: 1.5625–100 μg/mL) in LB medium. After 24 h incubation at 37 °C, the OD_600_ was measured.

### Growth curve analysis

To verify whether baicalin influences the growth of APEC-O78 and *V. harveyi* BB170, the bacterial growth curve was examined after cultivating in the presence of baicalin, and the APEC-O78 or *V. harveyi* BB170 alone as control. The concentrations of baicalin were selected from 12.5–50 μg/mL. In brief, the APEC-O78 was added into 50 mL of LB medium and *V. harveyi* BB170 was added into 50 mL of AB medium. Then, baicalin and APEC-O78 or *V. harveyi* BB170 were co-cultured at 37 °C  or 30 °C, and the value of OD_600_ was measured using a plate reader at 1 h intervals for 7 h.

### Assay for AI-2 activity of *V. harveyi* BB170 and APEC-O78

The AI-2 bioassay was performed as described previously^26^ with some modifications. After baicalin was co-cultured with APEC-O78 in DMEM at 37 °C for 4 h, cell-free culture fluids were collected by centrifugation at 12,000 × g at 4 °C for 10 min, filtered by 0.22 μm filters and stored at −80 °C until subsequent analysis.

*V. harveyi* BB170, used as an AI-2 reporter strain, was diluted by 1:5000 in fresh AB medium and then 180 μL of the bacterial culture was mixed with 20 μL of the collected supernatants or diluted baicalin (12.5, 25 and 50 μg/mL). Then incubated in a black flat-bottomed 96-well plate (Corning Costar, Fisher Scientific, Canada) at 30 °C for 4 h. The DH5α supernatant was regarded as negative control and the *V. harveyi* BB152 supernatant detected as positive control. Bioluminescence was measured via BHP9504 microplate luminescence analyzer (Beijing Hamamatsu Photonics Technology Co., Beijing, China) and experiments were done in triplicate.

### Detection of biofilm formation

The biofilm formation was measured by a crystal violet straining method. Briefly, 150 μL of diluted baicalin was transferred to a 96-well microtiter plate with 50 μL bacterium solution (OD_600_ = 0.05) with incubation at 37 °C for 24 h. Then, the wells were washed gently three times with 200 μL of PBS, fixed with methanol for 15 min and stained with 200 μL of 0.1% crystal violet for 5 min at room temperature. The wells were then washed with 200 μL of sterile water three times and dried at 37 °C for 10 min, 150 μL of 33% glacial acetic acid was added to the wells to dissolve the crystal violet. The total biofilm cells were then measured at 570 nm.

### Expression of virulence genes and inflammatory genes

The transcriptional levels of APEC-O78 virulence genes and inflammatory genes of chicken type II pneumocytes were tested using qRT-PCR after incubating with baicalin (12.5, 25 and 50 μg/mL) for 4 h. The RNA was extracted by TRIZOL reagent according to the manufacturer’s instruction, and then 1 μg of RNA was reverse transcribed to cDNA by reverted first strand synthesis kit with oligo (dT) primers (Thermo Scientific, Waltham, MA, USA). The PCR reactions were performed using a 7500 real-time PCR system (Applied Biosystems, Foster, CA) with FastStart SYBR Green PCR Master Mix (Roche, Mannheim, Germany). The *rrsG* and ACTB gene were served as housekeeping genes and the untreated *E. coli* and cells as a reference sample. Primers for real-time PCR are listed in Table 1.

**Table 1:** Primers for qRT-PCR.

Primer name	Sequence (5′ to 3′)
*rrsG-F*	TATTGCACAATG GGCGCAAG
*rrsG-R*	ACTTAACAAACCGCCTGCGT
*LuxS-F*	ACGCCATTACCGTTAAGATG
*LuxS-R*	AGTGATGCCAGAAAGAGGGA
*Pfs-F*	CGGCAACAGCCAGGAACTCA
*Pfs-R*	GCGAAAATCCGCCACAACTT
*fyuA-F*	TTGGCGACCAGGGTAAGAGC
*fyuA-R*	AGACCCGCAGTAGGCACGAT
*csgA-F*	AGATGTTGGTCAGGG CTCAG
*csgA-R*	CGTTGT TACCAAAGCCAACC
*csgB-F*	AATCAGGCAGCCATAATTGG
*csgB-R*	CCATAAGCACCTTGCGAAAT
*LsrB-F*	AGCATCCTGGCTGGGAAATTGT
*LsrB-R*	AAATTCTTTCACCGTGCCGCGT
*LsrK-F*	TGGAAGGCAATCAAATAGCA
*LsrK-R*	GCATACACTCACACGCCAGT
*fimB-F*	GGAGATTCATCCGCACATGTT
*fimB-R*	TCCCATATTCGCCAAAGCA
*fimA-F*	ATCGTTGTTCTGTCGGCTCT
*fimA-R*	ACTGGTTGCTCCTTCCTGTG
*H-NS-F*	CCGAACGAACTGCTGAATAG
*H-NS-R*	TTACCTTGCTCATCCATTGC
*rpoS-F*	CTCAACATACGCAACCTG
*rpoS-R*	GTCATCAACTGGCTTATCC
*ACTB-F*	ATGTGGATCAGCAAGCAGGAGTA
*ACTB-R*	TTTATGCGCATTTATGGGTTTTGT
*TNFIP2-F*	TGACCAGATTGCTCCTGAAA
*TNFIP2-R*	TCGTACTTCTGCCATTTTGC
*IL1β-F*	GAAGTGCTTCGTGCTGGAGT
*IL1β-F*	ACTGGCATCTGCCCAGTTC
*IL-4F*	GGTCTCTTCCTCAACATGCG
*IL-4R*	AAACAGAGTCGCTGGTAAGC

### Culture of chicken type II pneumocytes

The isolation and culture of chicken type II pneumocytes were performed as described previously^11^. In brief, the lung tissues of 13-day-old chicken embryos (Jilin Academy of Agricultural Sciences, Changchun, China) were digested by 0.25% trypsin and 0.1% IV collagenase (Invitrogen-Gibco, Grand Island, NY, USA) for 10 min and 15 min, respectively, and then the total cells were attachment cultured for twice. Finally, the unattached cells were collected and diluted with 20% fetal bovine serum (FBS) DMEM to cell count for 2 × 10^6^ cells**/**mL. Cells were incubated into a 6-well plate for 18 h at 37 °C. The attaching cells on culture dish were chicken type II pneumocytes.

### Cell viability assay by MTT

The effect of baicalin on cell viability was evaluated using MTT colorimetric assay. Chicken type II pneumocytes were seeded in a 96-well plate (2 × 10^6^ cells**/**ml, 100 μL) and were treated with different concentrations of baicalin for 18 h. After treatment with MTT solution for 4 h, the supernatants were removed and the formazan produced was dissolved in 150 μL of DMSO per well. The plate was shaken for 10 min. Finally, resulting absorbance was measured at 570 nm using a Bio-Tek microplate reader (Bio-Tek Instruments Inc., Winooski, VT, USA).

### Lactate dehydrogenase (LDH) activity detection

After APEC-O78 infected chicken type II pneumocytes cells for 4 h with baicalin in a 24-well plate, the supernatants were collected after centrifugation at 300 × g for 5 min and 12,000 × g for 10 min. The LDH activity was determined according to the manufacturer’s protocol (Jiancheng Technology Co., Nanjing, China). LDH activity was calculated as follows: LDH activity (U/L) = (Aexp–Acon)/(Asta–Abla) × 0.2 × 1000, where Aexp is the absorbance of test samples, Acon is the absorbance of control samples, Asta standard hole, and Abla of blank wells.

### Adhesion assay

Confluent monolayers of chicken type II pneumocytes cells (10^6^ cells**/**well) were cultured with APEC-O78 suspensions and different concentrations of baicalin to obtain a multiplicity of infection (MOI) of 1:100 in a 6-well plate. The plate was incubated for 4 h at 37 °C and 5% CO_2_. Monolayers were then washed three times with PBS, then 400 μl of 1% Triton X-100 (Sigma, St. Louis, MO, USA) were added to 6-well plate and co-incubated for 5 min to disrupt the cells. Viable bacterial cells were determined by plating dilutions of the lysates onto agar.

### Measurement of cytokine production by ELISA

After treatment with APEC-O78 suspensions and baicalin (12.5, 25, 50 and 100 μg/mL) in 12-well plate, the culture supernatant was collected. Inflammatory cytokines TNF-α, IL-1β and IL-4 were detected using chicken ELISA kits (BPRO, Lengton Bioscience Co., Ltd., Shanghai, China) according to the instructions of the manufacturer. Finally, the absorbance of each sample was determined by a microplate reader and was calculated according to the standard curve.

### Western blot assay

Western blotting was used to determine the expression of NF-κB pathway. The cell samples were dissociated by RIPA lysis buffer (Beyotime Biotechnology, Jiangsu, China) supplemented with protease inhibitor mixture (Roche Applied Science, Indianapolis, USA) and centrifuged at 12,000 × g for 15 min at 4 °C. Then, the cell lysates were fractionated on 12% SDS-PAGE and transferred to PVDF membranes. These PVDF membranes were blocked with 5% BSA for 4 h and then incubated with special primary antibody (1:500 dilution) at 4 °C overnight. Afterwards, the membranes were incubated with the corresponding HRP labeled secondary antibodies (1:25,000 dilution) at room temperature for 2 h, and then washed three times using TBST buffer. The immunoreactive proteins were detected using an enhanced chemiluminescence western blotting detection kit. The *β*-actin protein served as an internal control.

### Statistical analysis

All results in this study were evaluated using one-way ANOVA in SPSS 13.0 software (SPSS Inc., Chicago, IL, USA). Data are presented as the means ± standard deviations (SDs) of at least three independent experiments. P values of less than 0.05 were considered statistically significant.

### Compliance with Ethical Standards

All the animal researches and facilities were carried out in accordance with the experimental practices and standards. All experiments comply with the manual of the care and use of laboratory animals published by the US National Institutes of Health. All experimental protocols were approved by the Institutional Animal Care and Use Committee of Jilin University (IACUC).

## Results

### The antibacterial effect of baicalin on APEC-O78

**Figure 2:**
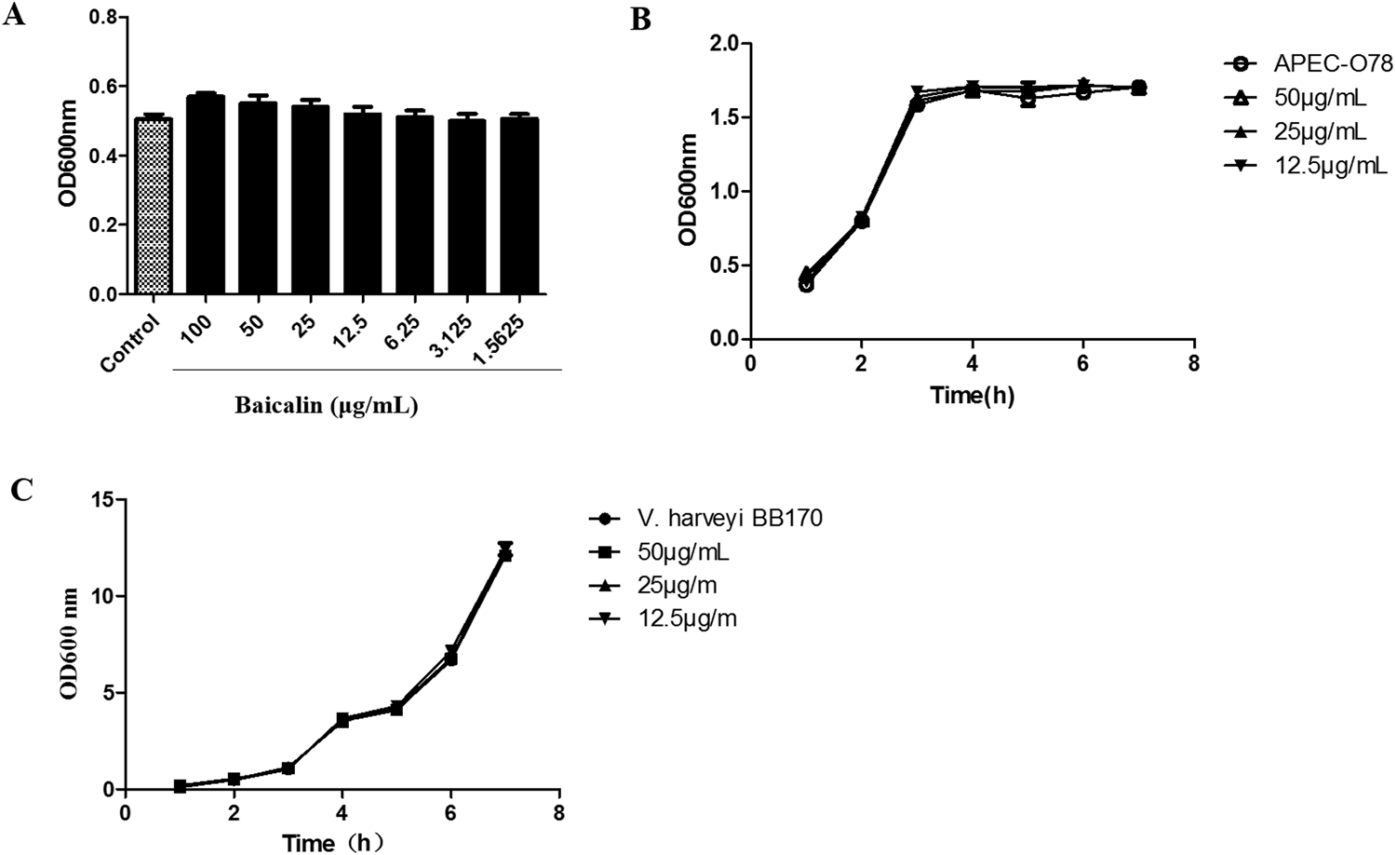
MIC of baicalin against APEC-O78 and growth curve of APEC-O78 and *V. harveyi* BB170 with baicalin. Baicalin (1.5625–100 μg/mL) incubated with APEC-O78 at 37 °C for 24 h, and then the OD_600_ was measured. (**B**) Baicalin (12.5, 25, 50 μg/mL) and APEC-O78 or (**C**) *V. harveyi* BB170 co-cultured at 37 °C, and then the value of OD_600_ of inocula was counted at 1 h intervals for 7 h.

We detected the MIC of baicalin and the growth curve of APEC-O78 to assess the antibacterial effect of baicalin on APEC-O78. As shown in Fig. 2A, baicalin (1.5625–100 μg/mL) had no inhibitory effect on the growth of APEC-O78. Therefore, the MIC of baicalin against APEC-O78 was >100 μg/mL. Furthermore, Fig. 2B,C showed that baicalin (12.5–50 μg/mL) had no effect on the growth curve of APEC-O78 and *V. harveyi* BB170.

### AI-2 Measurement

**Figure 3:**
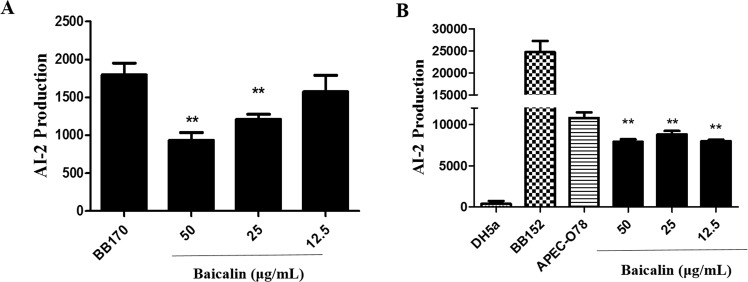
Measurement of AI-2 production. The different bacterial suspensions incubated with the reporter strain *V. harveyi* BB170. The AI-2 bioluminescence was examined. *p < 0.05 and **p < 0.01 are significantly different from the BB170 group or APEC-O78 group.

According to the above described measurement condition, the effect of baicalin on AI-2 production of *V. harveyi* BB170 and APEC was measured. As the Fig. 3A shows, the AI-2 reporter strain *V. harveyi* BB170, can produce the AI-2 in the process of growth. Baicalin (25 and 50 μg/mL) significantly inhibited the AI-2 production of *V. harveyi* BB170. Meanwhile, the data revealed that baicalin (12.5, 25 and 50 μg/mL) significantly reduced the AI-2 production compared with APEC in the absence of baicalin (Fig. 3B) and excluded the effect of baicalin on *V. harveyi* BB170.

### Biofilm formation

**Figure 4:**
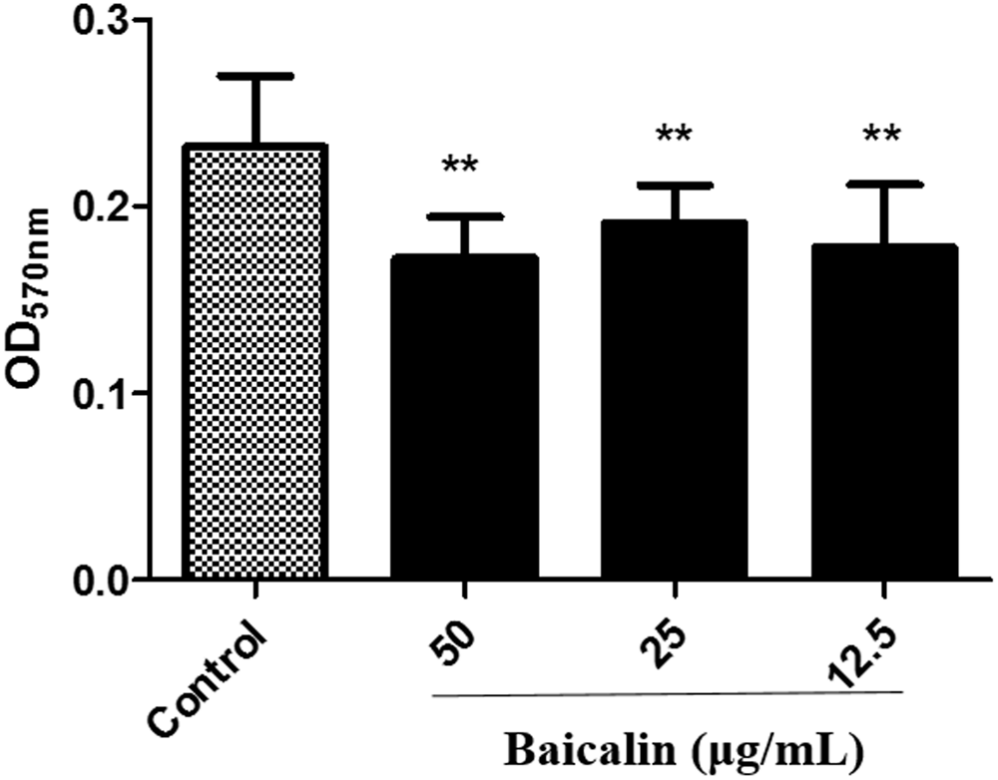
Biofilm formation. 12.5, 25, 50 μg/mL of baicalin and were incubated with APEC-O78 for 48 h, the biofilm formation was tested through a crystal violet straining method. *p < 0.05 and **p < 0.01 are significantly different from the control group.

The inhibition of APEC-O78 biofilm formation by the baicalin was measured using a crystal violet staining method. As a result, all three concentrations of baicalin had good potentials for disrupting APEC-formed biofilm. As shown in Fig. 4, baicalin (12.5, 25 and 50 μg/mL) was able to significantly reduce the biofilm formation.

### Expression of virulence genes

**Figure 5:**
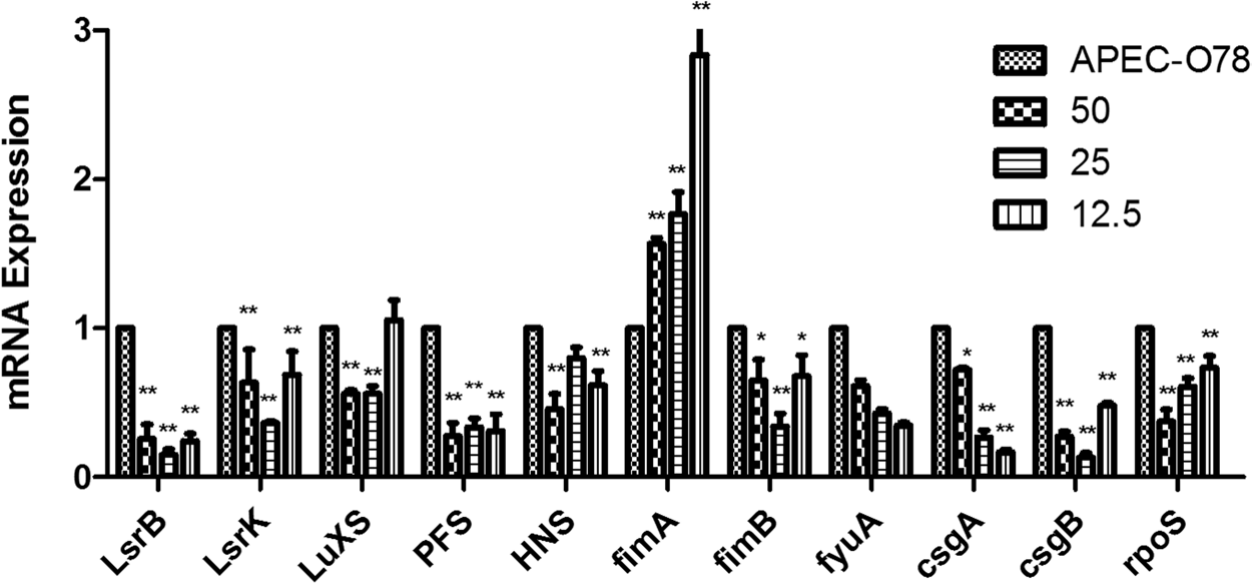
The Expression of virulence genes of APEC-O78. After APEC-O78 treated with 12.5, 25, 50 μg/mL baicalin, the expression of virulence genes of APEC-O78 was detected by qRT-PCR.

The qRT-PCR results (Fig. 5) demonstrates that the related virulence genes (*LsrB, LsrK, LuxS, PFS, HNS, fimB, fyuA, csgA, csgB, rpoS*) of APEC-O78 were decreased significantly except for the *fimA* gene after treatment with baicalin (12.5, 25 and 50 μg/mL).

### Cytotoxicity of baicalin to chicken type II pneumocytes

**Figure 6:**
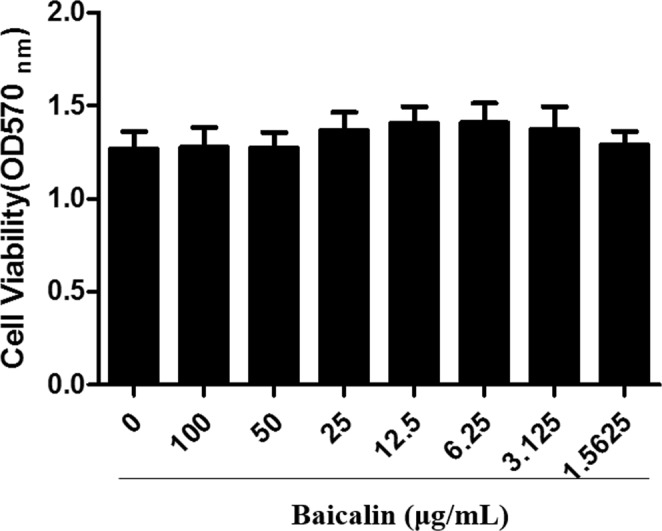
Cytoxicity of baicalin on chicken type II pneumocytes. Cells were treated with 1.5625–100 μg/mL baicalin for 18 h. The cytoxicity was evaluated using MTT assay.

Whether baicalin had a toxic effect on chicken type II pneumocytes was analyzed by MTT assay. The results in Fig. 6 showed that baicalin (1.5625–100 μg/mL) had no effect on cell viabilities of chicken type II pneumocytes.

### Effect of baicalin on APEC-infected chicken type II pneumocytes

**Figure 7:**
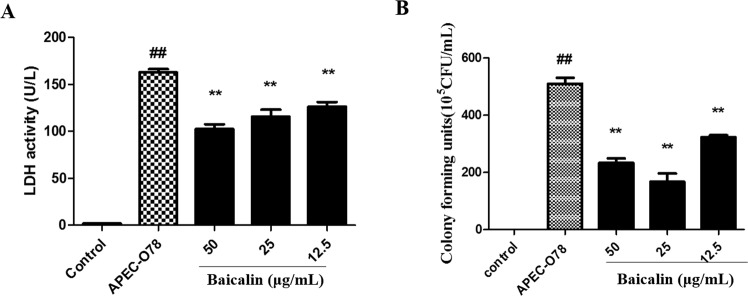
LDH activity examination and adhesion assay. (**A**) APEC-infected chicken type II pneumocytes treated with baicalin for 4 h. Then suspension was collected for LDH examination. (**B**) The supernatants above diluted and viable bacterial cells were determined by plating dilutions of the lysates onto agar. ##*p* < 0.01 is significantly different from the control group; *p < 0.05 and **p < 0.01 are significantly different from the APEC-O78 group.

We explored the protective effect of baicalin on APEC-infected chicken type II pneumocytes by examining the count of APEC-O78 adherent on cells and LDH release of APEC-infected cells. The examination of LDH revealed that 12.5–50 μg/mL of baicalin significantly reduced the LDH release of chicken type II pneumocytes infected by APEC-O78 (Fig. 7A). Furthermore, bacterial adhesion assay exhibited that 12.5, 25 and 50 μg/mL of baicalin significantly inhibited the adhesion of APEC-O78 on chicken type II pneumocytes (Fig. 7B).

### Effect of baicalin on APEC-induced inflammatory cytokine production

**Figure 8:**
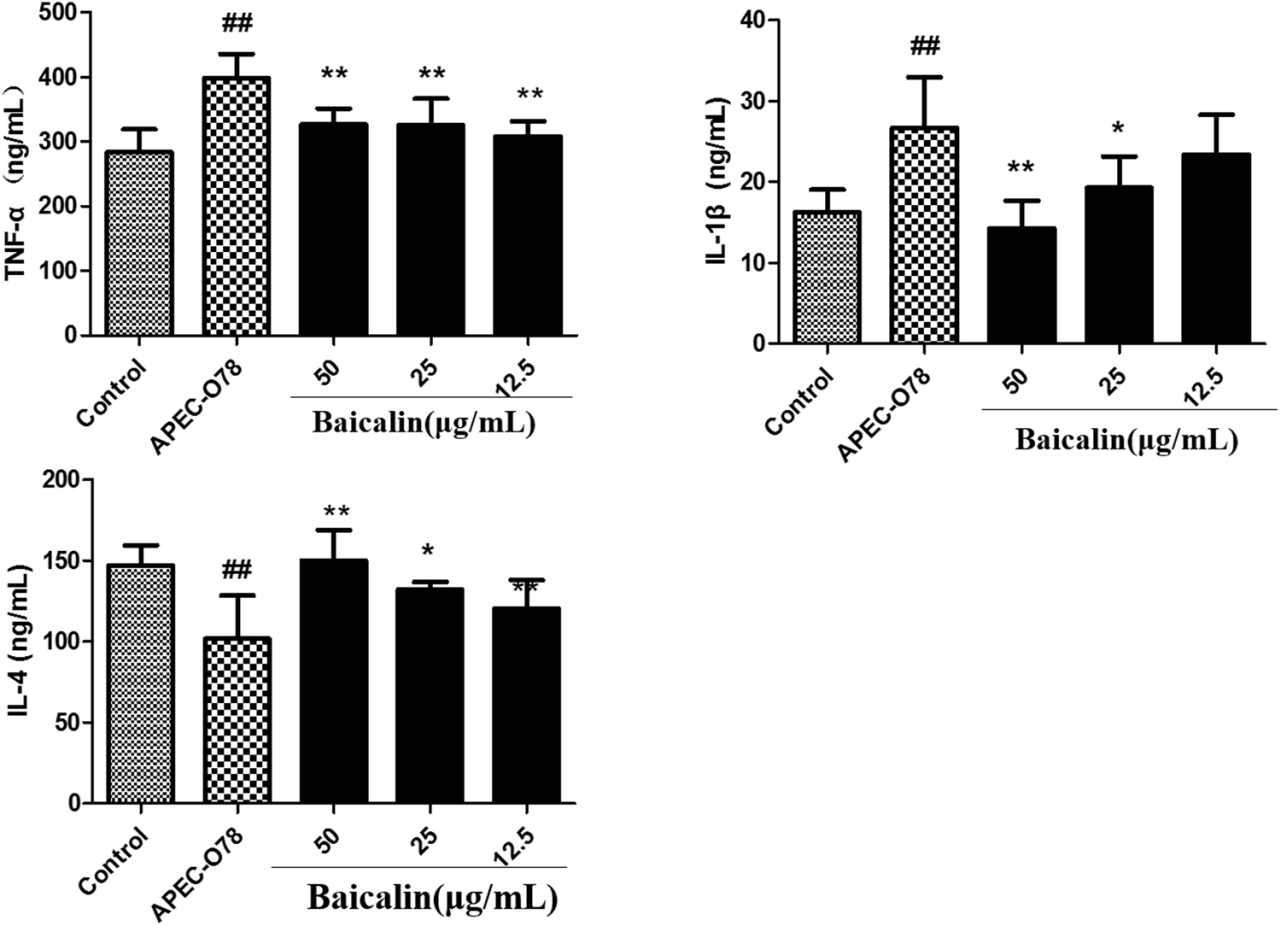
The Effect of baicalin on APEC-induced inflammatory cytokine production. After the cells exposed to APEC-O78 and baicalin(12.5, 25, 50 μg/mL), extracellular levels of TNF-α (**A**), IL-1β (**B**), and IL-4 (**C**) were measured using ELISA kits. ##*p* < 0.01 is significantly different from the control group; *p < 0.05 and **p < 0.01 are significantly different from the APEC-O78 group.

**Figure 9:**
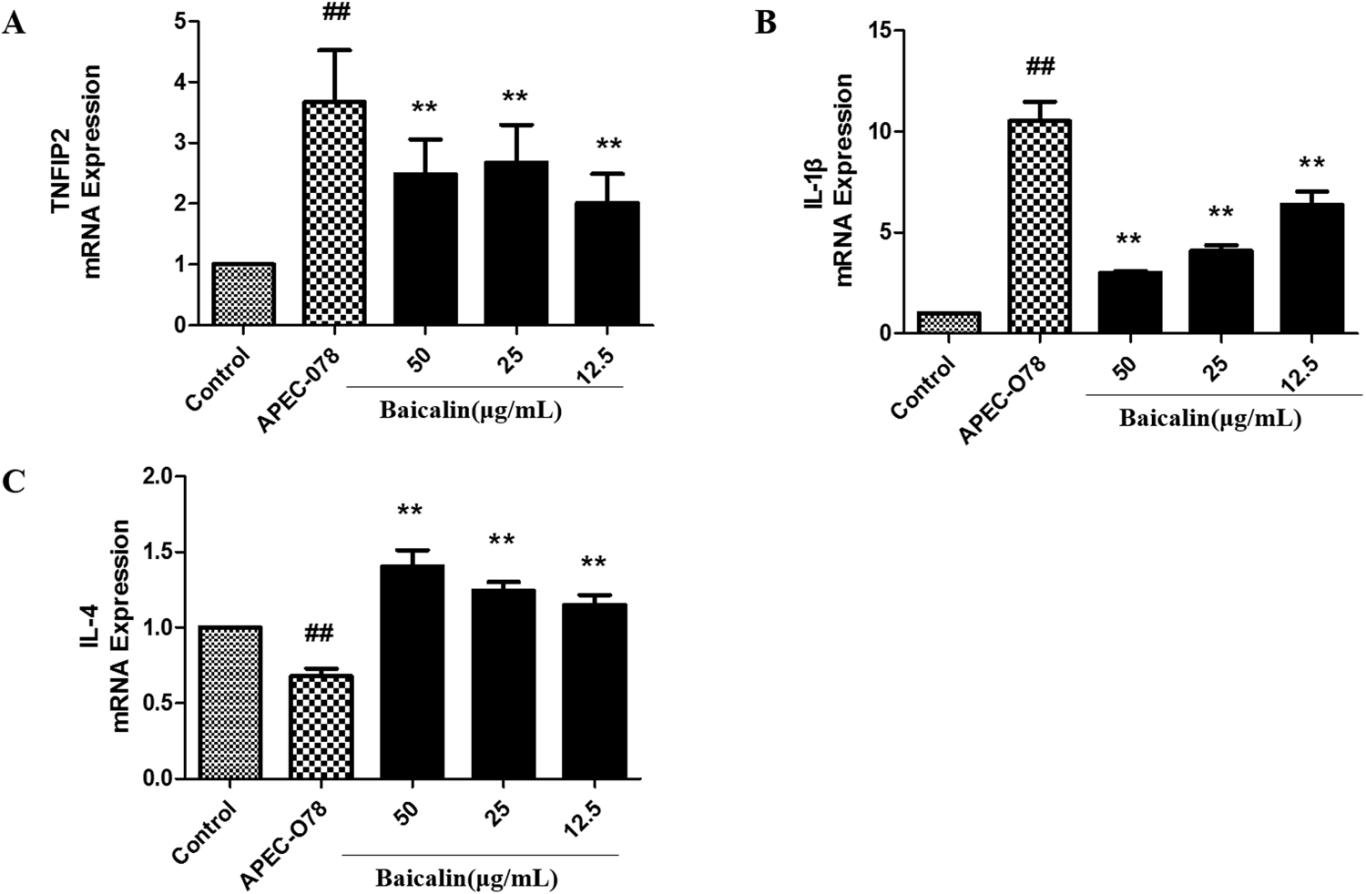
The mRNA expression of inflammatory genes after treatment with baicalin. Total RNA was extracted and reverse transcribed to cDNA, the mRNA expression of TNFIP2 (**A**), IL-1β (**B**), and IL-4 (**C**) were detected by qRT-PCR. ##*p* < 0.01 is significantly different from the control group; *p < 0.05 and **p < 0.01 are significantly different from the APEC-O78 group.

The levels of inflammatory cytokines TNF-α, IL-1*β*, and IL-4 were tested by ELISA. As shown in Fig. 8A–C, baicalin significantly inhibited the expression of pro-inflammatory cytokines, TNF-α and IL-1β, and increased the levels of anti-inflammatory cytokines IL-4 induced by APEC-O78. In addition, the gene expression of TNF-α, IL-1β, and IL-4 were consistent with the results of ELISA (Fig. 9A–C).

### Effect of baicalin on APEC-induced NF-κB signaling pathway

**Figure 10:**
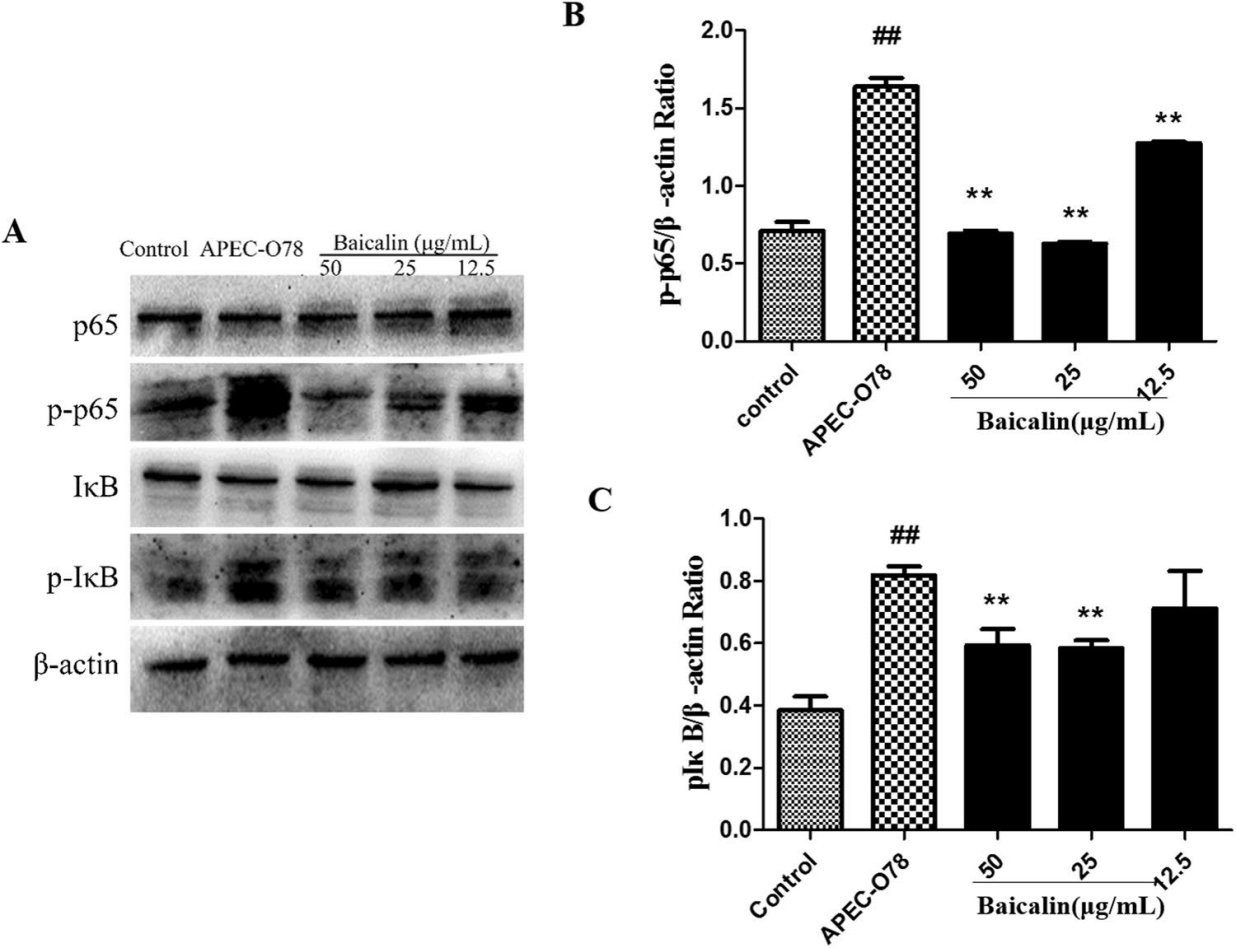
Effects of baicalin on NF-*κ*B pathway activation by APEC-O78. Chicken type II pneumocytes were treated with baicalin and infected by APEC for 4 h, and then the protein levels were analyzed by western blotting (**A**). Finally, the blots were semi-quantified (**B**,**C**). ##*p* < 0.01 is significantly different from the control group; *p < 0.05 and **p < 0.01 are significantly different from the APEC-O78 group.

The NF-κB signaling pathway plays an important role in the production of inflammatory cytokines. In order to test the protective mechanism of bacalin on APEC-induced inflammatory response, the expression of NF-κB signaling pathway proteins were tested in the present study. The results showed that the expression of phosphorylated NF-κB p65 and phosphorylated IκB were markedly increased in the APEC-O78 group compared with the control group. However, baicalin significantly inhibited the expression of phosphorylated NF-κB p65 and phosphorylated IκB induced by APEC-O78 (Fig. 10).

## Discussion

In order to handle with bacterical infections, the effect of baicalin on QS of APEC-O78 and inflammatory response induced by APEC-O78 were tested in the present study. We have demonstrated that baicalin is able to inhibit QS, biofilm formation and the expression of virulence genes. In addition, the results showed that baicalin treatment significantly increased the levels of IL-4, as well as inhibited the levels of TNF-α and IL-1β though suppressing the activation of NF-κB signaling pathway.

QS is adjusted by auto-inducers which are small chemical signals to regulate a wide variety of cellular activities, such as motility, virulence, antibiotic production, biosurfactant formation, and biofilm formation^27^. AI-2 is produced by the activated methyl cycle by the AI-2 synthase Pfs and LuxS^28^. LsrB, along with other proteins that form the Lsr transport system, acts as the substrate binding protein of an ATP binding cassette (ABC) transport system responsible for AI-2 internalization. Once inside the cell, AI-2 is phosphorylated by the kinase LsrK^29^. It has been reported that the AI-2 secretion influences the rpoS and H-NS expression when the bacteria encounter different environment stress^30^. RpoS and H-NS control the expressions of many genes related to cellular responses to a variety of changes of environmental conditions and genes related to biofilm formation^31–33^. In this study, our results showed that baicalin markedly decreased the *rpoS* and *H-NS* gene expression and biofilm formation. Hence, in this study, baicalin may reduce the *rpoS* and *H-NS* expression of APEC-O78 by inhibiting the AI-2 production. The high pathogenicity of APEC is involved in many virulence factors including adhesion, iron-binding, proteins, antiserum factors, outer membrane proteins, and hemolysis, such as fimA and fimB (type 1 pili), fyuA (iron uptaking), csgA and csgB (curly pili A), and so on^8^. In the present study, baicalin reduced the *fimB*, *fyuA*, *csgA* and *csgB* gene expression. The *fimB* is related in the type 1 fimbriae which are the major bacterial adherence factor to both biotic and abiotic for *E. coli*. The *fyuA*, as a structural gene in the core region of the high pathogenicity island, influence the iron uptake capacity of APEC and the expressions of some virulence factors. Tu^34^ has proved that *irp2* and *fyuA* knockout APEC strain has weakened transcription of virulence genes and capacity to adhere to DF-1 cells. As our data shows, baicalin can effectively suppress the adhesion of APEC-O78 to chicken type II pneumocytes. Therefore, baicalin attenuated the adhesion capacity of APEC-O78 by inhibiting the virulence genes expression. Curli fimbria is known to be associated with bacterial biofilm formation and the UPEC-4 ▵csgA mutant lost its adherence to HTB-9 but continued to adhere to the HUVEC and Vero cells^35^. The inhibitory effect of baicalin on APEC-O78 biofilm formation and adhesion capacity may be concerned with curli fimbria gene - *csgA* and *csgB*. However, this assumption needs more evidence to verify. In this study, baicalin significantly decreased the biofilm formation, AI-2 secretion and virulence genes expression, consistent with the suppression of the LDH release of chicken type II pneumocytes induced by APEC-O78.

The inflammatory response is important for defense against the pathogenic invasion. TNF-α, and IL-1β are the main pro-inflammatory cytokines^36^. IL-4 and IL-10, on the other hand, are the main anti-inflammatory cytokines and considered to have ability to inhibit the production of pro-inflammatory cytokines^37,38^. In the APEC-O78 induced inflammatory injury of chicken type II pneumocytes, the expression of TNF-α and IL-1β were increased, and the production of IL-4 and IL-10 were reduced^11^. Furthermore, the production of pro-inflammatory cytokines is required for the activation of NF-κB signaling pathway activation^39–41^. In the present study, the results showed that baicalin treatment significantly increased the levels of IL-4, as well as inhibited the levels of TNF-α and IL-1β though suppressing the activation of NF-κB signaling pathway induced by APEC-O78 in chicken type II pneumocytes.

In conclusion, baicalin reduced cell damage of APEC-infected chicken type II pneumocytes through interfering with QS, biofilm formation, and the expression of virulence genes. Meanwhile, baicalin inhibited the release of inflammatory cytokines through alleviating the activation of NF-κB signaling pathway induced by APEC-O78. Our study suggested that baicalin may be a potential therapeutic agent for the treatment of APEC induced colibacillosis.

## Data Availability

All data generated or analysed during this study are included in this published article (and its Supplementary Information files).
